# Mixing Performance of a Passive Micromixer Based on Split-to-Circulate (STC) Flow Characteristics

**DOI:** 10.3390/mi15060773

**Published:** 2024-06-10

**Authors:** Makhsuda Juraeva, Dong-Jin Kang

**Affiliations:** School of Mechanical Engineering, Yeungnam University, 280 Daehak-ro, Gyoungsan 38541, Republic of Korea; mjuraeva@ynu.ac.kr

**Keywords:** split-to-circulate, submerged circular wall, degree of mixing (DOM), saddle point, flow impingement, concave wall, convex wall

## Abstract

We propose a novel passive micromixer leveraging STC (split-to-circulate) flow characteristics and analyze its mixing performance comprehensively. Three distinct designs incorporating submerged circular walls were explored to achieve STC flow characteristics, facilitating flow along a convex surface and flow impingement on a concave surface. Across a broad Reynolds number range (0.1 to 80), the present micromixer substantially enhances mixing, with a degree of mixing (DOM) consistently exceeding 0.84. Particularly, the mixing enhancement is prominent within the low and intermediate range of Reynolds numbers (0.1<Re<20). This enhancement stems from key flow characteristics of STC: the formation of saddle points around convex walls and flow impingement on concave walls. Compared to other passive micromixers, the DOM of the present micromixer stands out as notably high over a broad range of Reynolds numbers (0.1≤Re≤80).

## 1. Introduction

In recent years, various microfluidic devices [1,2,3] have been broadly applied in the industry of biomedical diagnostics, chemical analysis, food and genetic engineering, drug delivery, and medicine. These devices serve multiple purposes, from analyzing samples to delivering drugs, and micromixers play a pivotal role in these systems by homogenizing the sample reagents on a microscale. Accordingly, micromixers are designed to achieve key objectives: minimizing reagent consumption, faster mixing process, and maintaining compact size [3,4]. To meet these objectives, ongoing research is focused on various micromixer designs capable of rapidly and efficiently mixing in microscale dimensions, enhancing the overall performance of microfluidic systems.

In spite of ongoing research efforts, mixing in a microfluidic system continues to face issues stemming from limitations imposed by molecular diffusion [2,3]. The microscale dimensions and sluggish flow velocity within micromixers result in low Reynolds numbers, leading to inefficient mixing. To innovate new technologies that can overcome these inherent obstacles is an urgent necessity. The burgeoning development of the microfluidic industry depends heavily on highly efficient micromixers. Although diverse efforts have been employed to tackle these challenges, the field still lacks novel mechanisms to achieve truly efficient mixing [1,2,3,4].

Designs aimed at enhancing mixing in micromixers can be categorized as either passive or active. Most active micromixers rely on an additional energy source to achieve enhanced mixing by disrupting fluid flow and generating circulatory flows. Acoustic fields [5], electric current [6], magnetic fields [7], thermal energy [8,9], or pulsating flow [10,11] are commonly used as an energy source. Even though active micromixers offer the advantage of forcefully controlling flow, they also come with certain drawbacks, including higher consumption of energy, complex structure, and challenges in fabrication [2,5]. These limitations significantly impede active micromixers from being applied in microfluidic systems, particularly in the context of portability and cost-effectiveness [12].

On the contrary, passive micromixers employ geometric rearrangements to induce circulatory flow, without any moving components or additional energy sources. This approach simplifies integration, making it cost-effective and adaptable in various microfluidic systems. Various geometric configurations have been proposed to generate circulatory flows, including channel wall twisting [13], staggered herringbone [14], block in the junction [15], split-and-recombine (SAR) [16,17,18], surface grooves and baffles [19,20], modified Tesla structure [21,22], convergent–divergent micromixer [23], mixing unit stacking in the transverse direction [24,25], and submerged structures [26,27,28]. Meanwhile, it is noteworthy that most passive micromixers result in mixing enhancement over a limited range of Reynolds numbers.

The need for rapid mixing times, typically in the millisecond order, for biology and chemistry applications has promoted a micromixer capable of effective operation over a broad range of Reynolds numbers (Re < 100) [29,30,31,32,33]. Within this spectrum of Reynolds numbers, micromixing is controlled by two different mechanisms: chaotic convection and molecular diffusion. Accordingly, micromixing can be divided into three regimes based on the dominating mechanism: molecular diffusion dominant, transitional, and convection dominant. Among these regimes, mixing performance in the transitional regime is found to be worst, with the corresponding Reynolds number range of approximately 0.5 to 10. Therefore, a new design concept is imperative to overcome these issues and achieve enhanced mixing performance over a broad range of Reynolds numbers.

In the transitional mixing regime, both molecular diffusion and chaotic convection play equally significant roles, necessitating geometric modifications that take into account both mixing mechanisms. Typically, in the diffusion dominant regime, mixing enhancement involves creating an elongated interface between the two reagents. To materialize this mixing characteristics, one typical design is to place various types of obstacles inside the microchannel. For example, Cheri et al. [34] studied four different obstacle geometries to obtain mixing enhancement at a low Reynolds number of 0.1. Hsiao et al. [27] mounted rectangular winglet pairs on the bottom wall of the main channel of a T-shaped micromixer, optimizing the winglet geometry to enhance mixing. Ortega-Casanova [35] introduced a heaving square cylinder to maximize the mixing at very low Reynolds numbers. Conversely, in the regime of convection dominance, mixing improvement is primarily achieved through the presence of circulating flow such as vortices [36]. For example, Li et al. [37] proposed dislocating the sub-channels of a SAR micromixer to enhance mixing. Ansari et al. [38] introduced the design concept of unbalanced collision on a SAR micro-mixer, inducing Dean vortex of different intensity in the subsequent channels. Hong et al. [39] promoted the Coanda effect by modifying the Tesla micromixer, allowing the reagent to follow the curved surfaces and thus enhancing transverse dispersion. Raza et al. [36] recommended a modified Tesla structure for the applications in the intermediate (1 < Re ≤ 40) and high Reynolds number ranges (Re > 40). Makhsuda et al. [26] demonstrated that submerged planar structures enhance mixing performance in the range of Re ≥ 5, with two Dean vortices bursts to promote mixing performance.

As these geometry modifications are typically focused on either high or low Reynolds numbers, they often result in poor performance in the other regime of Reynolds numbers. One typical approach to address this issue is to combine two different geometric features. For example, Sheu et al. [40] combined the Dean vortex and two fluids collision effects using tapered-and-curved microchannels, resulting in mixing enhancement in the range of Re ≥ 5. Raza et al. [41] achieved mixing indices greater than 90% for the Reynolds numbers Re ≥ 20, combining unbalanced SAR and baffles. Bazaz et al. [42] investigated various geometric modifications such as obstruction, teardrop, pillar, nozzle, and Tesla. They demonstrated that combining planar geometric modifications is a practical strategy enhancing mixing performance. Subsequently, many researchers are focusing on planar structure modifications to induce 3D flow characteristics, without additional complexities associated with conventional 3D micromixers.

The submerged planar structures have proved their efficacy in significantly enhancing the mixing performance, especially in the transitional regime of mixing. This design concept has an additional advantage of reducing associated pressure drop. For instance, Makhsuda et al. [26] demonstrated that submerged planar mixing cells result in 182% improvement in the degree of mixing (DOM) and 44% reduction in the associated pressure drop at Re = 10 due to secondary vortices in the cross-flow direction. Hsiao et al. [27] achieved an enhancement in DOM over a broad range of 0.125≤Re≤64, using submerged pairs of winglets in a microchannel. Various microfabrication techniques like Xurography [43] and a single-step dual-layer photolithography [44] can be used to easily fabricate passive micromixers like the present one. The Xurography method employs thin, double-sided adhesive films, allowing for simple tailoring of submerged structures using a cutter plotter. For example, Martínez-López et al. [45] applied the Xurography method in the fabrication of a passive micromixer.

In this paper, we propose a new design concept called split-to-circulate (STC), which promotes circulatory flows after flow split. To analyze the mixing efficacy of STC, three different designs were investigated. Each design consists of four mixing units, with each unit comprising multiple split-to-circulate flow passages built by circular walls. The proposed micromixer was assessed by computing the DOM at the outlet and the associated pressure drop.

## 2. Governing Equations and Computational Procedure

We used ANSYS^®^ Fluent 2021 R2 [46] to simulate the mixing process in the present micromixer. The governing equations include the 3D Navier–Stokes equation, the continuity equation, and a species convection–diffusion equation. Since the flow is laminar, the following equations are employed:

(1) $$\left(\vec{u}\cdot \nabla \right)\vec{u}=-\frac{1}{\rho }\nabla p+\nu \nabla^{2}\vec{u}$$

(2) $$\nabla \cdot \vec{u}=0$$

where $\vec{u}$, *p*, and *ν* are the velocity vector, pressure, and kinematic viscosity, respectively. The evolution of mixing is simulated by solving a convection–diffusion equation:

(3) $$\left(\vec{u}\cdot \nabla \right)\varphi =D\nabla^{2}\varphi$$

where *D* and *φ* represent the mass diffusivity and mass fraction of a fluid, respectively.

The commercial software ANSYS^®^ FLUENT 2021 R2 [46] utilizes the finite volume method to solve the governing equations. The convective terms in the governing Equations (1) and (3) were discretized using the QUICK (quadratic upstream interpolation for convective kinematics) scheme, a discretization scheme of third-order accuracy. At the two inlets, inlet 1 and inlet 2, the velocity distribution was assumed to be uniform, while the outflow condition was applied at the outlet. As the Knudsen number *Kn* is less than 10^−3^, the no-slip boundary condition was specified along all walls. Here, the Knudsen number is defined as the ratio of the mean free path length of fluid molecules to a characteristic length of the micromixer. Fluid A and fluid B were assumed to be injected through inlet 1 and inlet 2, respectively. Therefore, the mass fraction of fluid A is *φ* = 1 at inlet 1 and *φ* = 0 at inlet 2.

The mixing performance of the present micromixer was assessed in terms of DOM and mixing energy cost (MEC). DOM is defined as follows:

(4) $$DOM=1-\frac{1}{\xi }\sqrt{\sum_{i=1}^{n}\frac{{\left(\varphi_{i}-\xi \right)}^{2}}{n}},$$

where *φ_i_* and *n* are the mass fraction of fluid A in the *i*th cell and the total number of cells, respectively. *ξ* = 0.5 represents the state of complete mixing of two fluids. When the two fluids are completely mixed, DOM is 1. DOM = 0 indicates no mixing. MEC measures the effectiveness of present micromixer in the following form [35,47]:

(5) MEC=Δpρumean2DOM×100,

where $u_{mean}$ is the average velocity at the outlet, and Δp is the pressure load between the inlet and the outlet.

The fluid properties, including diffusion coefficient, density, and viscosity, were assumed to be the same as those of water. They are *D* = 1.0 × 10^−10^ m^2^s^−1^, *ρ* = 997 kg/m^3^, and *ν =* 0.97 × 10^−6^ m^2^s^−1^, respectively. The Schmidt (Sc) number, which is the ratio of the kinetic viscosity to mass diffusivity of the fluid, is approximately 10^4^. The Reynolds number was defined as $Re=\frac{\rho U_{mean}d_{h}}{\mu }$, where $\rho ,\;{{\;d}_{h},\;U}_{mean},\;and\;\mu$ indicate the density, the hydraulic diameter of the outlet channel, the mean velocity at the outlet, and the absolute viscosity, respectively.

## 3. Validation of the Numerical Study

For high Schmidt (Sc) number simulations, the accuracy of simulated results can be compromised by numerical diffusion. Several strategies have been devised to enhance numerical accuracy. Examples include employing particle-based simulation methodologies such as the Monte Carlo method [48], lattice Boltzmann equation [49], and reducing cell Peclet number for grid-based methods. The cell Peclet number $Pe_{c}$ is defined as $Pe_{c}=\frac{U_{cell}l_{cell}}{D}$, with $U_{cell}$ and $l_{cell}$ indicating flow velocity and cell size, respectively. A recommended practice is to restrict the cell Peclet number to $Pe_{c}\leq 2$, as suggested by Bayareh [50]. However, these strategies entail substantial computational costs, rendering numerical studies impractical for studies like the present one. A practical alternative is to perform a grid independence test and validate the results with the corresponding experimental data [51]. Instead of resorting to any computationally intensive remedies, this paper adopted a pragmatic approach commonly employed in many numerical studies.

**Figure 1:**
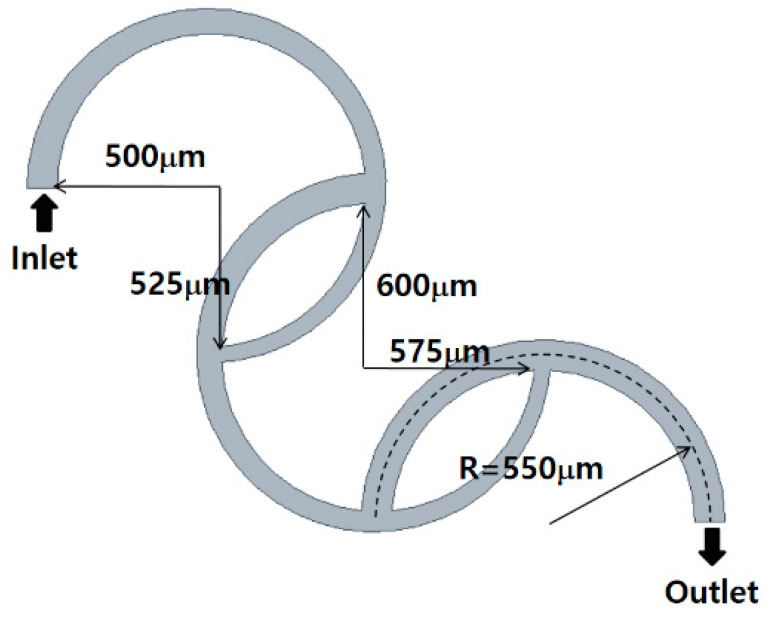
Diagram of the SAR examined by Sheu et al. [40].

The SAR micromixer examined by Sheu et al. [40] was utilized to validate the current numerical approach. Figure 1 illustrates a schematic diagram, which comprises three ring-shaped channels. The first ring channel is connected to the second ring channel at 180° apart from the inlet, and the second ring channel is connected to the third ring channel in a similar way. The length of the first two ring channels on the inlet side is three-quarters, while the last ring on the outlet side is two-quarters long. The center line of all three channels has the same radius of 550 μm. The cross-section of the inlet and outlet is a square of 100 μm length. The width of the first two ring channels on the inlet side tapers from 100 μm to 50 μm, while its depth remains constant at 100 μm. Conversely, the cross-section of the third channel on the outlet side remains unchanged, and it is a square of 100 μm long.

Sheu et al. [40] evaluated the mixing performance defining the mixing index (MI) in the following form:

(6) $$MI=1-\frac{\sigma_{D}}{\sigma_{D,o}}$$

and

(7) $$\sigma =\sqrt{\frac{1}{n}\sum_{i=1}^{n}{\left(\varphi_{i}-\varphi_{ave}\right)}^{2}}$$

where $\sigma_{D}$ is the standard deviation on a cross-section normal to the flow. $\sigma_{D,o}$ is the standard deviation at the inlet, and $\varphi_{ave}$ is the average value over the sampled section.

**Figure 2:**
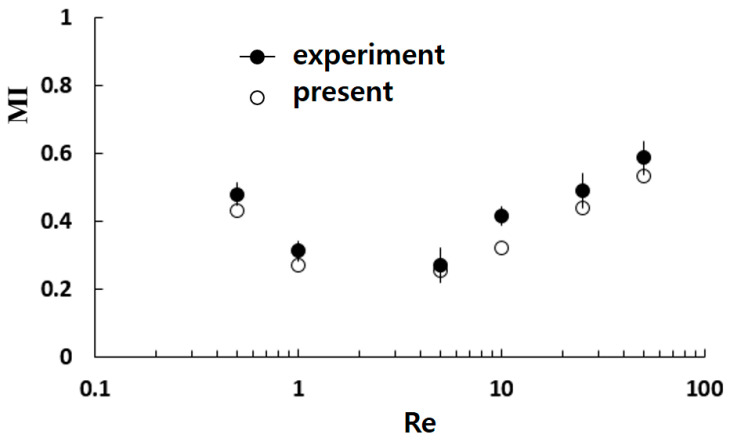
Validation of the numerical approach: experiment by Sheu et al. [40].

Figure 2 compares the simulation results with the corresponding experimental data by Sheu et al. [40]. The present simulation accurately predicts the mixing index (MI) variation with Reynolds number. Even though there are some discrepancies between the numerical solutions and experimental data. This discrepancy is due to several factors, including numerical diffusion and experimentation uncertainty.

## 4. Present Micromixer Based on Split-to-Circulate Flow Characteristics

**Figure 3:**
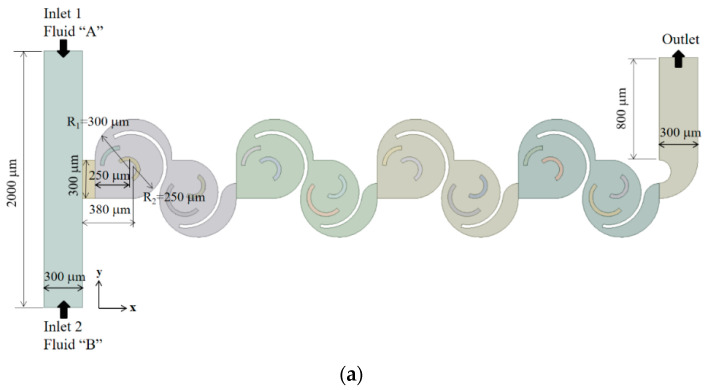

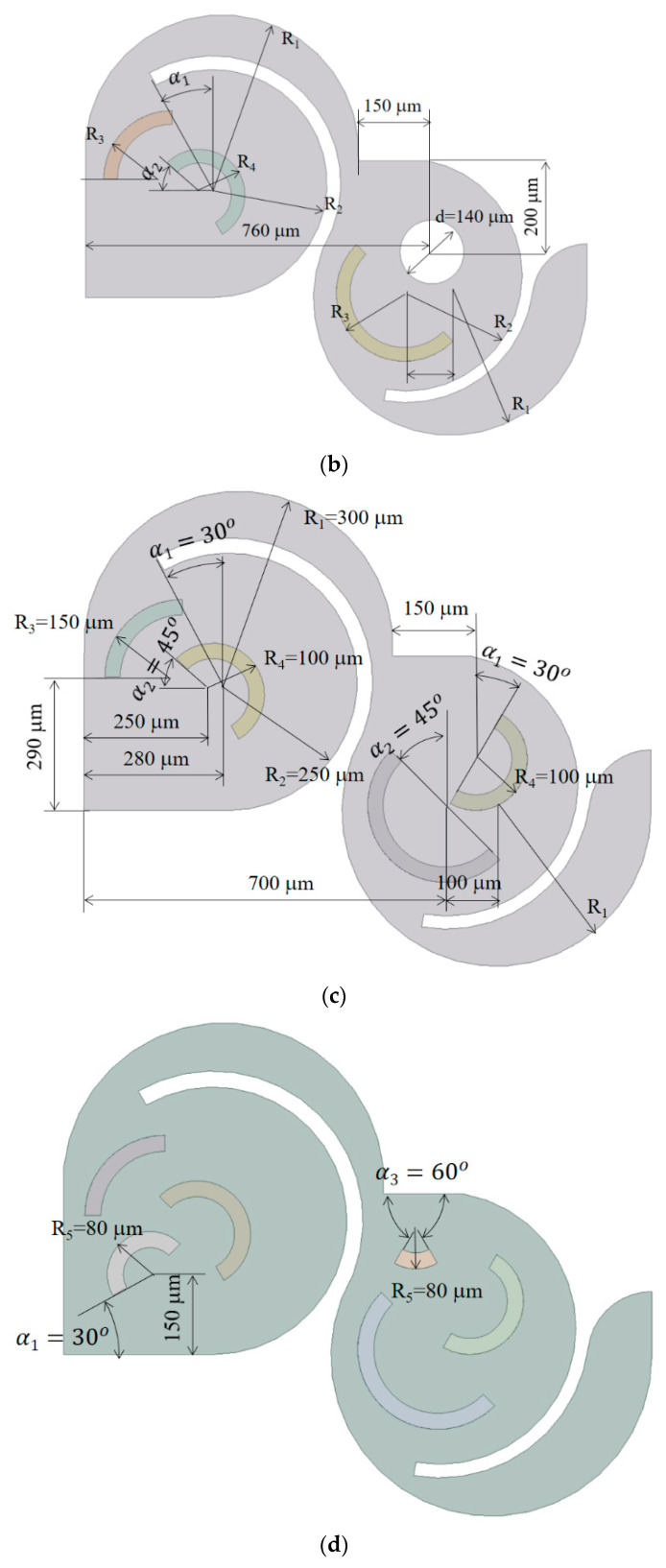

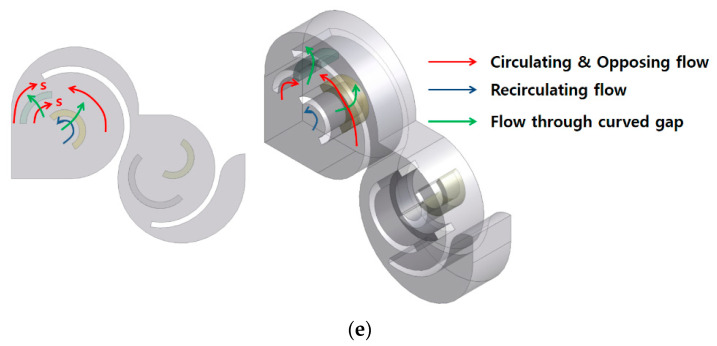
Schematic diagram of present micromixer: (**a**) front view, (**b**) mixing unit of Case 1, (**c**) mixing unit of Case 2, (**d**) mixing unit of Case 3, and (**e**) flow patterns within a mixing unit of Case 1.

The present micromixer consists of four mixing units, as illustrated in Figure 3a. Each mixing unit contains two mixing cells with circular outer boundaries. These mixing cells house multiple circular flow passages, each characterized by distinct radii denoted as R_1,_ R_2,_ R_3_, and R_4_. Additionally, two circular walls with radii R_3_ and R_4_ serve as baffles, both with a height of 200 μm, which is shorter than the overall height of the micromixer. As a result, the circular passages along the walls of radii R_3_ and R_4_ facilitate the flow crossover of two separate flow passages by traversing the passage walls in the radial direction.

In this paper, three distinct designs were simulated to assess the effectiveness of micromixers utilizing STC principles, as depicted in Figure 3: Case 1, Case 2, and Case 3. Case 1 incorporates a SAR (split-and-recombine) geometry in the second mixing cell, while Case 2 exclusively employs the STC (split-to-circulate) concept, as illustrated in Figure 3c. The SAR geometry is constructed using a circular cylinder with a radius of 70 μm. Meanwhile, for Case 3, the number of STCs is increased from two to three to promote more flow circulation. To achieve that, the flow is split once more by a circular wall at the entrance of each mixing cell.

Figure 3b–d provides a schematic diagram of the three cases. For example, Case 2 exhibits three circular passages guiding the fluid to flow in both clockwise and counterclockwise directions. Each passage was intentionally designed with distinct radii, denoted as R_1_, R_2_, R_3_, and R_4_. This specific mixing unit was engineered to promote circulatory flows guided by circular walls, as visually demonstrated in the figure. The anticipated flow patterns in the first mixing cell are depicted in Figure 3e. The first characteristic involves a recirculating flow along a concave wall, illustrated as the blue line in Figure 3e. The second pattern entails an impingement of two opposing flows depicted as red in Figure 3e, resulting in a saddle point (denoted as “s” in the figure). The third pattern promotes flow crossover submerged circular walls, represented as green in Figure 3e. These flow patterns significantly enhance mixing performance across a broad range of Reynolds numbers. For Case 1, one circular wall in the second mixing cell is replaced with a circular cylinder, as illustrated in Figure 3c. Therefore, any difference in results between Case 1 and Case 2 may demonstrate the efficacy of the circular wall as a flow splitter. For Case 3, the number of flow splits is increased from two to three, compared with Case 2. Therefore, any deviation in results may suggest whether an optimal number of STCs within a mixing cell exists.

The cross-section of the inlet and outlet branches is a rectangle, measuring 300 μm wide and 200 μm deep. Both inlet 1 and inlet 2 are 1000 μm long, while the outlet branch measures 800 μm in length. The two inlets are positioned on opposite sides so that the mixing process primarily evolves in the subsequent mixing channel. The axial length of all the mixing units is approximately 4.2 mm.

**Figure 4:**
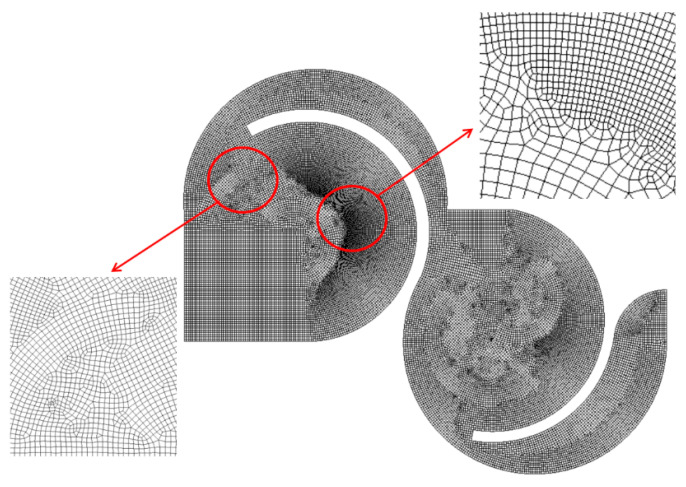
Example of mesh in a mixing unit.

The micromixer depicted in Figure 3a was meshed with a sufficient number of cells. In order to mitigate potential numerical diffusion, the cell size was carefully decided through preliminary simulations. These simulations were carried out at Re = 0.5 for Case 1. In the generation of mesh, the edge size was limited to below a certain value. In this test, the limit was varied from 4 μm to 6 μm, corresponding to cell numbers ranging from 2.14 × 10^6^ to 10.6 × 10^6^. Figure 4 presents a magnified view of the mesh within a mixing unit. According to Okuducu et al. [52], the cell type can largely affect numerical accuracy. Hexahedral cells are strongly recommended over prism and tetrahedral cells. Consequently, hexahedral cells were mostly employed in the mesh, as illustrated in Figure 4. It has a limited number of prism cells, while tetrahedral cells were completely avoided.

The convergence index (GCI) [53,54] was used to assess the uncertainty of simulation results. The GCI is calculated using the following formula:

(8) $$GCI=F_{s}\frac{\left|\varepsilon \right|}{r^{p}-1},$$

where *F_s_*, *p,* and *r* indicate the safety factor of the method, the order of accuracy of the numerical method, and the grid refinement ratio, respectively. ε is determined as follows:

(9) $$\varepsilon =\frac{f_{coarse}-f_{fine}}{f_{fine}},$$

where *f_coarse_* and *f_fine_* are the numerical solutions obtained with a coarse and fine grid, respectively. *F_s_* was set at 1.25, following the recommendation of Roache [53]. The edge size limit was varied as 4 μm, 5 μm, and 6 μm, corresponding to cell counts of 3.4 × 10^6^, 5.8 × 10^6^, and 10.9 × 10^6^, respectively. Upon computing the GCI with three different limits of edge size, the GCI is approximately 2.4% with the edge size limit of 5 μm. Therefore, 5 μm was selected for meshing the computational domain, considering its favorable GCI value and a balance between numerical accuracy and computational cost.

## 5. Mixing Performance of Present Micromixer

The mixing performance of the present micromixers was simulated across a broad range of Reynolds numbers, spanning from 0.1 to 80. For these simulations, a velocity ranging from 0.21 mm/s to 0.17 m/s was applied uniformly at the two inlets, resulting in volume flow rates ranging from 1.5 μL/min to 1206 μL/min. The evaluation of mixing performance involves calculating the degree of mixing (DOM) at the outlet, along with the required pressure drop.

**Figure 5:**
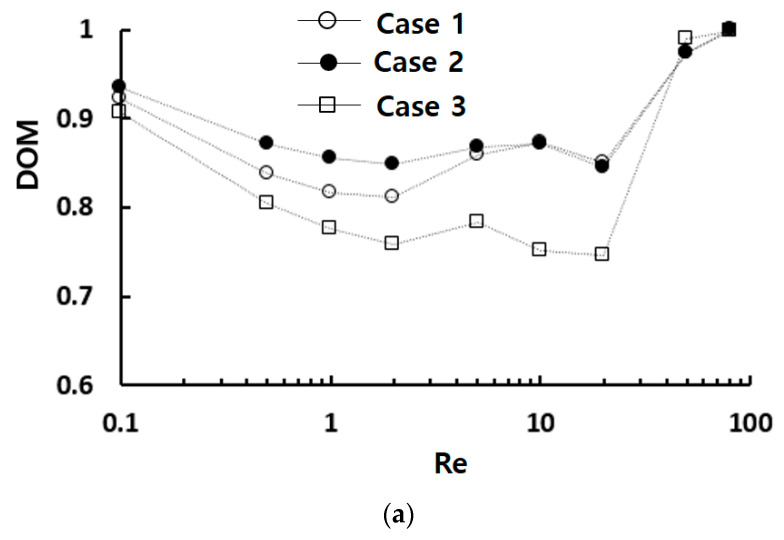

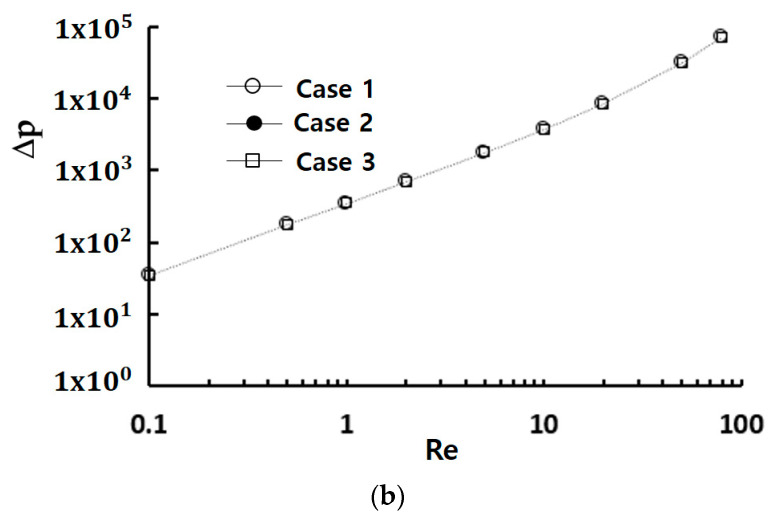
Comparison of three designs: (**a**) DOM vs. Re and (**b**) Δ*p* vs. Re.

Figure 5 compares three designs in terms of DOM and required pressure drop. In Figure 5a, Case 2 demonstrates the best DOM performance across the entire range of Reynolds numbers. The DOM of Case 2 is substantially higher than that of Case 3 for Re<50. This result suggests that the number of STCs can be optimized in terms of DOM; an excessive number of STCs could block the fluid stream and diminish mixing performance. Meanwhile, the DOM of Case 2 is higher than that of Case 1 for Reynolds numbers Re≤5, while there is no noticeable difference between them for Re ≥ 5. This demonstrates that STC is more effective than SAR in achieving higher DOM in the molecular diffusion dominant and transitional regime of mixing. However, the required pressure drop shows negligible dependence on the geometric variation, as demonstrated in Figure 5b.

**Figure 6:**
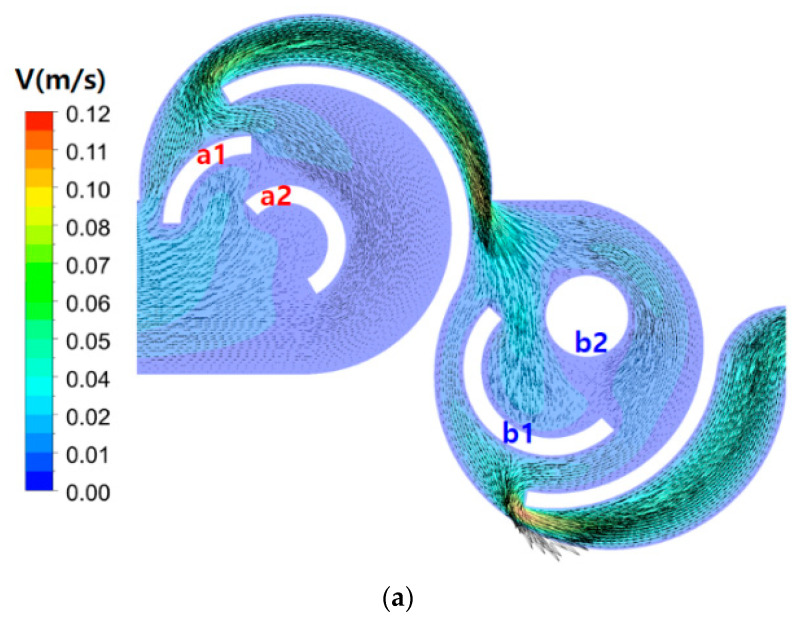

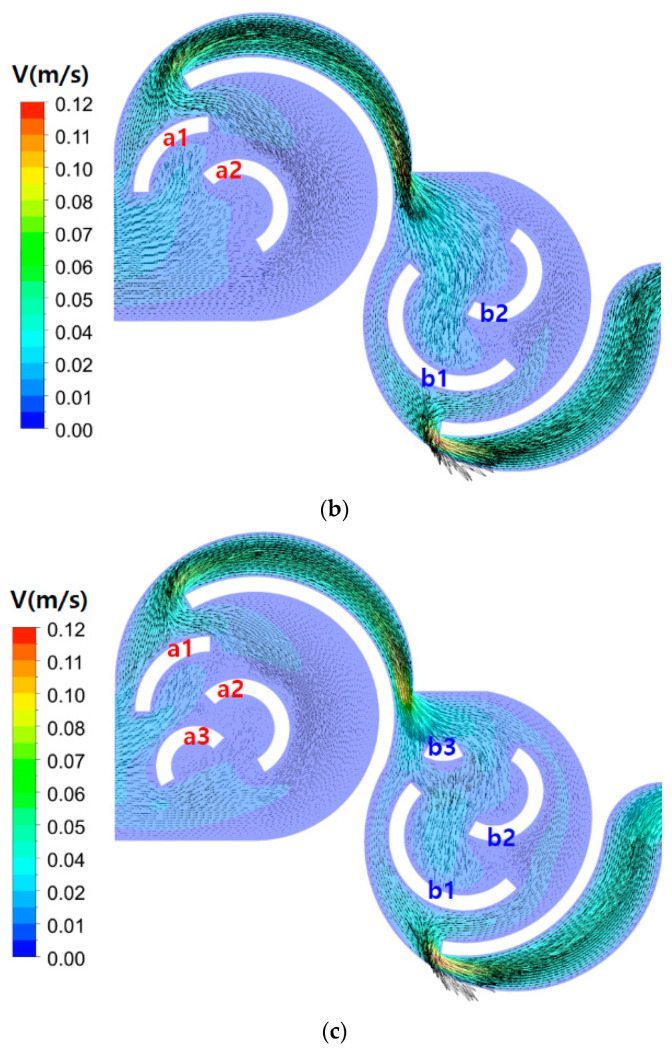
Saddle points within the first mixing unit at Re = 2: (**a**) Case 1, (**b**) Case 2, and (**c**) Case 3.

Figure 6 demonstrates how the three designs affect mixing flow at Re = 2, within the first mixing unit. The figures present the contours of the concentration of fluid A on the mid-plane in the z-direction. For Case 1, two saddle points in each mixing cell are observed as expected. The saddle points, a1, a2, and b1, indicate that the split flows by a submerged circular wall, follows along the convex wall, and forms a saddle point. At each saddle point, the two split flows originate from circular walls of different radii. Therefore, the collision at a saddle point is asymmetric. Meanwhile, the saddle point b2 is formed by a circular cylinder embedded instead of a circular wall of Case 2, and the recombination of two flows is symmetric, showing the weakest circulatory motion among the four saddle points. Additionally, the flow entering the second mixing cell impinges on the two concave walls in Case 2, resulting in additional mixing enhancement. This result suggests that the submerged circular wall could play a better role as a flow splitter within a mixing cell, resembling a bucket shape. Conversely, for Case 3, two more saddle points, a3 and b3, are formed, as shown in Figure 6c. These two additional saddle points do not lead to any noticeable enhancement in mixing performance, as additional flow split weakens chaotic mixing around the circular wall. This result suggests that the number of submerged structures within a mixing unit could be optimized in terms of DOM.

**Figure 7:**
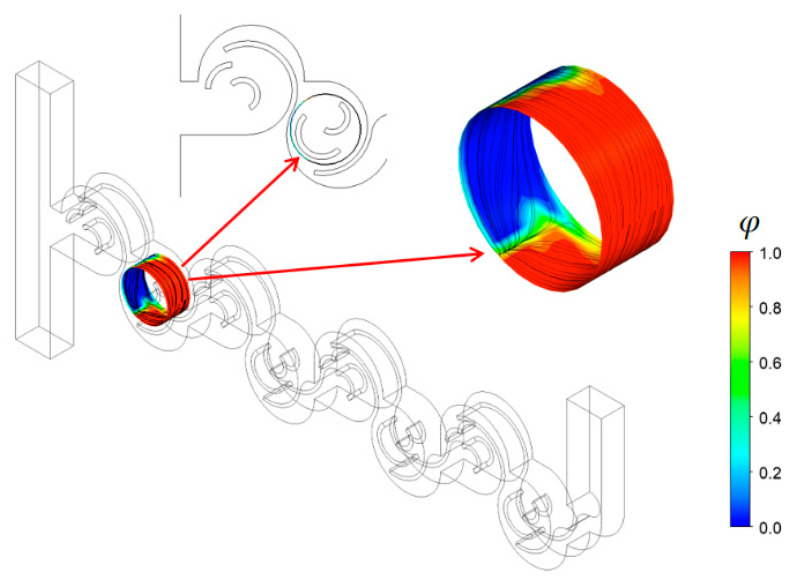
Mixing on a cylindrical surface passing the saddle point “b1” at Re = 2; “b1” is noted in Figure 6b.

Figure 7 demonstrates how mixing occurs around a saddle point at Re = 2. The cylindrical surface passes through the saddle point “b1” described in Figure 6b. We can observe a vigorous mixing as the two fluids (red and blue in the figure) merge along a line, but it is asymmetric as they originate from circular walls of different radii. The submerged circular wall causes an additional mixing on the right-hand side of the cylindrical surface.

**Figure 8:**
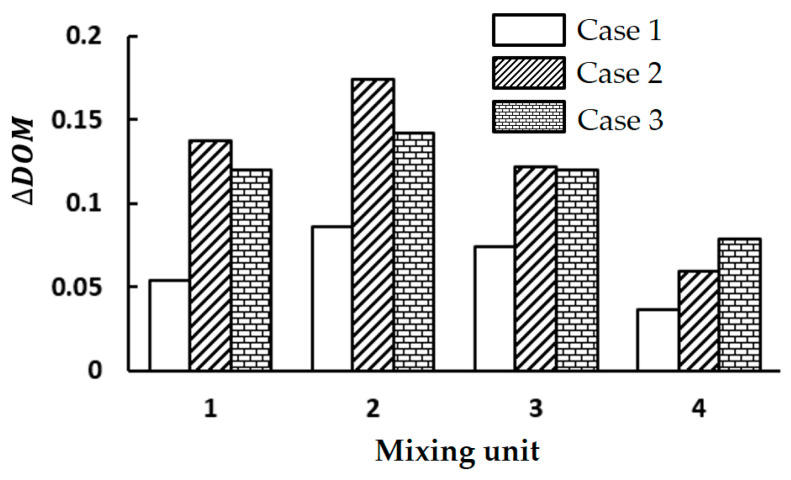
Comparison of DOM increment in each mixing unit at Re = 2.

Figure 8 compares the DOM increment of three cases in each mixing unit at Re = 2. It confirms that the mixing cell design of Case 2 performs the best, especially in the first and second mixing units. The efficacy of the mixing unit in terms of DOM decreases after the second mixing unit, irrespective of its design. The DOM increment of Case 2 is noticeably higher than that of Case 1 throughout the entire mixing units. This enhancement suggests that the submerged circular wall would be a better flow splitter than a circular cylinder within a mixing cell. This is because the circular wall resembles a bucket shape and promotes fluid impingement on concave walls. The increment of Case 2 is also higher than that of Case 3, up to the third mixing unit.

**Figure 9:**
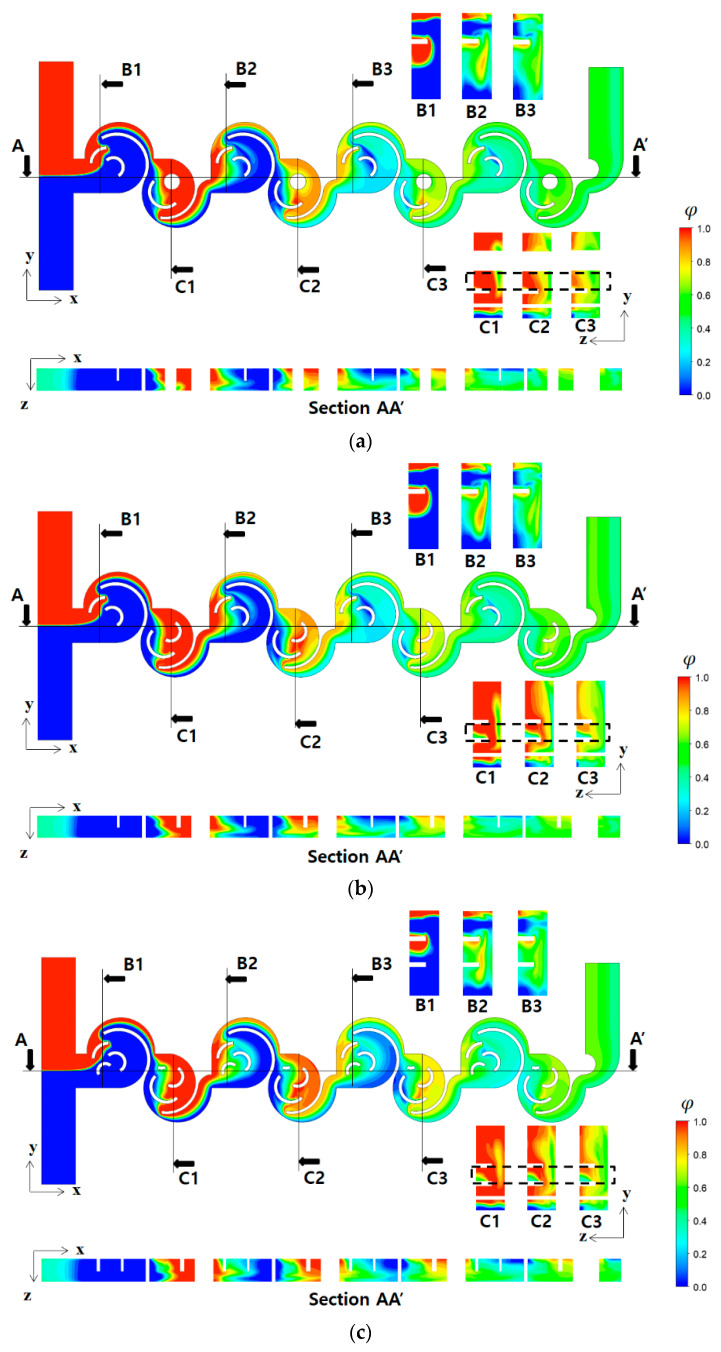
Evolution of mixing along the micromixer at Re = 10: (**a**) Case 1, (**b**) Case 2, and (**c**) Case 3.

Figure 9 illustrates how the flow characteristics described in Figure 6 affect the mixing process in the present micromixer in terms of concentration contours at various cross-sections at Re=2. All the cross-sections, B1, B2, B3, C1, C2, and C3, are obtained at a specific location as indicated, normal to the x-direction. Comparing the concentration contours at the cross-sections, B1, B2, and B3 of the three cases, there is negligible difference among them. This suggests that any geometric difference in the first mixing cell contributes negligibly to mixing enhancement. Conversely, the cross-sections C1, C2, and C3 of the three cases demonstrate distinct differences among them. The contours inside the dotted line box show the greatest difference between Case 1 and Case 2 (or Case 3). The contours of Case 2 and Case 3 show more vigorous mixing compared with Case 1; the green spot in the box indicates more active mixing. Case 2 shows the widest green spot in the dotted line box; green means complete mixing (φ=0.5). These results suggest that the flow impingement on the concave wall could be a potential flow pattern to enhance mixing. This flow characteristic is obtained as the flow entering the second mixing cell encounters a concave surface of the circular wall resembling a bucket shape, as described in Figure 6.

**Figure 10:**
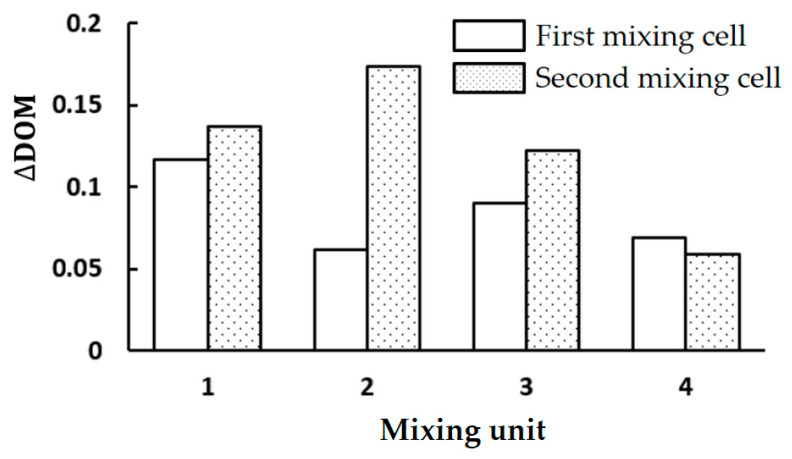
DOM increment in each mixing unit of Case 2 at Re = 2.

Figure 10 provides a comprehensive comparison of the DOM increment in each mixing cell of Case 2 at Re = 2. The second mixing cell of the second mixing unit contributes the most, and the DOM increment decreases as it goes further downstream. The last mixing unit contributes approximately 15% of the whole DOM. This implies that an additional mixing unit of more than four would not be cost-effective in enhancing mixing. Another finding is that the increment of DOM is higher in the second mixing cell than in the first mixing cell, except in the fourth mixing unit. The DOM increment in the second mixing cell of the second mixing unit is 1.8 times that of the first mixing cell. This confirms that the bucket shape arrangement in the second mixing cell results in additional mixing enhancement.

**Figure 11:**
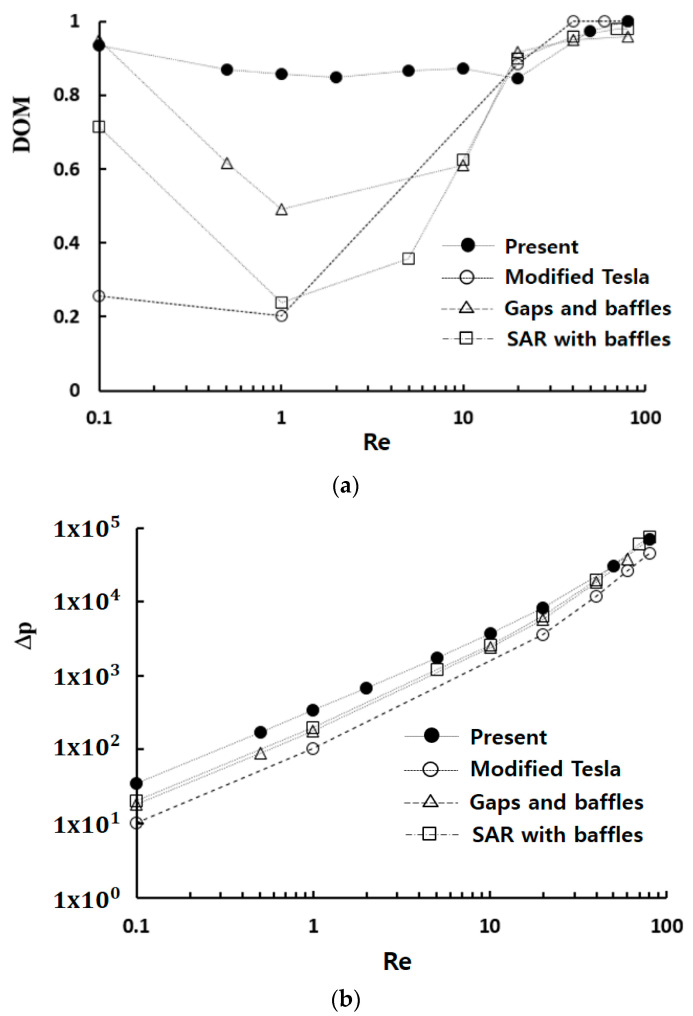
Comparison of the mixing performance of the present micromixer with other passive micromixers: (**a**) DOM vs. Re and (**b**) Δ*p* vs. Re.

Figure 11 provides a comparative evaluation of the mixing performance of Case 2 with other passive micromixers as a function of the Reynolds number: a modified Tesla micromixer [36], a passive micromixer with gaps and baffles [55], and a SAR micromixer with baffles [41]. All four passive micromixers underwent research under similar boundary conditions and physical properties. The present micromixer shows a significant enhancement of DOM in the low and intermediate range of Reynolds numbers (0.1<Re<20) compared to other passive micromixers. Through the entire Reynolds number range, the DOM of Case 2 is larger than 0.84. Specifically, the DOM of Case 2 is approximately 0.86 at Re = 1, while the modified Tesla micromixer [36], the passive micromixer with gaps and baffles [55], and the SAR micromixer with baffles [41] are 0.2, 0.24, and 0.49, respectively. This corresponds to 75% higher than that of the passive micromixer with gaps and baffles [55]. This result suggests that STC could serve as a promising design element for enhancing mixing performance in the molecular diffusion and transition regime. However, it should be noted that this enhancement is accompanied by an increase in the required pressure drop, as depicted in Figure 6b.

## 6. Conclusions

In this paper, we introduced a novel passive micromixer based on the STC (split-to-circulate) flow characteristics and thoroughly investigated its mixing performance. The present micromixer comprises four mixing units, each housing two mixing cells containing submerged circular walls that create several circular flow passages. Therefore, the number of submerged circular walls and its arrangement are key design parameters. To ascertain the significance of these design parameters, we have simulated three distinct designs using ANSYS^®^ Fluent 2021 R2 for computational analysis of mixing performance.

The present micromixer shows a significant enhancement of DOM within the low and intermediate range of Reynolds numbers (0.1<Re<20) compared to other passive micromixers, such as a modified Tesla micromixer, a passive micromixer with gaps and baffles, and a SAR micromixer with baffles. Moreover, the DOM of the present micromixer is larger than 0.84 across the entire range of Reynolds numbers (0.1≤Re≤80). These results prove that STC could be a potential element to obtain high mixing performance throughout a wide range of Reynolds numbers.

The mixing enhancement, particularly in the transition regime of mixing, is primarily attributed to two key flow characteristics facilitated by the submerged circular walls. Firstly, these walls split the flow and guide it along convex walls, leading to the formation of saddle points where flows originating from different radii mix vigorously. Secondly, the bucket-shaped configuration of the circular walls induces flow impingement on concave walls, further enhancing mixing. This underscores the superior effectiveness of submerged circular walls over conventional solid geometries like circular cylinders in enhancing mixing performance. These key flow characteristics are closely related to several geometric parameters such as radii and angle of circular walls. They can be optimized further to achieve an additional enhancement of DOM, and this is a topic of future research.

The STC design has demonstrated its effectiveness in enhancing the mixing performance of passive micromixers. Compared to other passive micromixers, the present design results in a notable enhancement in DOM, particularly within the low and intermediate Reynolds number range of 0.1<Re<20. Furthermore, the DOM of the present micromixer remains consistently high, surpassing 0.84 over a broad range of Reynolds numbers (0.1≤Re≤80), despite its simple geometry, which is comparable to other planar micromixers.

## Data Availability

The original contributions presented in the study are included in the article, further inquiries can be directed to the corresponding author.
